# Transfer of a Serial Representation between Two Distinct Tasks by Rhesus Macaques

**DOI:** 10.1371/journal.pone.0070285

**Published:** 2013-07-31

**Authors:** Greg Jensen, Drew Altschul, Erin Danly, Herbert Terrace

**Affiliations:** Department of Psychology, Columbia University, New York, New York, United States of America; Utrecht University, The Netherlands

## Abstract

Do animals form task-specific representations, or do those representations take a general form that can be applied to qualitatively different tasks? Rhesus monkeys (*Macaca mulatta*) learned the ordering of stimulus lists using two different serial tasks, in order to test whether prior experience in each task could be transfered to the other, enhancing performance. The *simultaneous chaining* paradigm delivered rewards only after subjects responded in the correct order to all stimuli displayed on a touch sensitive video monitor. The *transitive inference* paradigm presented pairs of items and delivered rewards when subjects selected the item with the lower ordinal rank. After learning a list in one paradigm, subjects’ knowledge of that list was tested using the other paradigm. Performance was enhanced from the very start of transfer training. Transitive inference performance was characterized by ‘symbolic distance effects,’ whereby the ordinal distance between stimuli in the implied list ordering was strongly predictive of the probability of a correct response. The patterns of error displayed by subjects in both tasks were best explained by a spatially coded representation of list items, regardless of which task was used to learn the list. Our analysis permits properties of this representation to be investigated without the confound of verbal reasoning.

## Introduction

Animal cognition is no longer an oxymoron. During the last 50 years, hundreds of experiments have shown that animals can solve problems by using representations of events that are not physically present during test, the *sine qua non* of cognitive processing [1]. In human participants, transfer of serial representations can be mediated by verbal and logical rules, but such rules can obscure underlying cognitive functions. Since neither language nor deliberative reasoning are available to rhesus monkeys [2], their performance on transfer tasks affords a more direct examination of those mechanisms.

Consider, for example, an experiment in which naïve rhesus monkeys were presented with the four adjacent pairs of items from an ordered set of alternatives, A, B, C, D, and E. Whenever a subject selected the earlier item in each pair, a reward was delivered. Subjects were then presented with non-adjacent pairs to test whether they could make a *transitive inference* (TI), such as “A comes before C,” on the basis of their previous training with AB and BC. Non-human primates were highly proficient at this task, such that their performance was indistinguishable from that of 4- and 6-year-old human children [3], [4]. This similarity raised two questions: How does a primate (human or otherwise) encode the information required to make this inference, and how similar are the serial representations used by non-human primates to those used by humans?

One clue suggesting a common mechanism is the *symbolic distance effect* (SDE), a ubiquitous phenomenon in studies of TI, as well as in other forms of serial reasoning [5]–[8]. Given an ordered list of items, judging the ordering of items is most difficult when the items have adjacent list positions, but becomes easier as the distance between items increases. For example, the SDE predicts that, all else being equal, the pair BE should be easier than the pair BD, which in turn should be easier than BC. If serial distance effects are an inherent property of the serial representation of list items, subjects should transfer those effects from one serial task to another, so long as the ordinal position of list items do not change.

The cognitive interpretation of these results is that subjects generate an implicit linear ordering of the full implied list (e.g. ABCDE), even though only two list items appear on each trial [9]. However, critics have correctly noted that humans can use logical and semantic tools to assist in the transfer of ordinal knowledge from one TI problem to another [10]. Those tools make it difficult to assess any underlying non-verbal representations of list items. Studying TI and its corresponding SDE in animals negates that criticism.

Most studies of serial learning in animals, including those that seek to explain SDEs, rely on an “associative” approach that eschews representative phenomena [11]–[13]. Serial learning has also been studied using computational simulations of neural networks inspired by neuroanatomy [14], [15]. Although these approaches to serial learning are not mutually exclusive and can indeed complement one another [16], [17], associative and computational accounts also share substantial weaknesses. Experiments studying TI typically use a very narrow range of experimental methods in which extensive training of adjacent pairs is followed by testing on non-adjacent pairs [13]. The resulting analytic focus on the ‘test’ phase provides very little information about initial learning. Instead, the models that this approach has produced are tailored to their test paradigms and are unable to account for performance in serial tasks other than “train adjacent,” followed by “test non-adjacent” at a later time. In order to overcome this limitation, we employed two paradigms that allowed us to examined learning on a trial-by-trial basis.

The first of these paradigms was the *simultaneous chain* (SimChain) task [18]. On each trial, subjects were presented with arbitrary photographic stimuli, denoted as A, B, C, D, & E. To earn a reward, subjects had to touch each item in the correct order, A
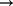
B
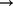
C
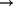
D
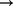
E. The physical locations of items in the SimChain were scrambled on each trial to ensure that subjects didn’t simply learn lists as motor sequences.

Our second task was a variation of the traditional TI paradigm. Only two stimuli were presented during each trial, and a food pellet was delivered whenever a subject selected the item with the earlier list position. Throughout training, subjects were shown all pairs (sampled randomly without replacement) rather than only adjacent pairs. We call this a *counterbalanced* TI task because exposure to each pair of items was uniform across the session, even as trials themselves were randomized. This method ensured that exposure to all pairs was consistent with respect to session time, which allowed learning for every pair to be examined in parallel with the others. Overall, our novel approach permitted a finer-grained analysis than traditional methods of training that presented only adjacent pairs.

Previous studies have shown that knowledge of serial order learned using one task can transfer to a distinctive serial task [8], [19]–[22]. Such demonstrations provide evidence that both tasks rely on the same representation. However, due to lengthy training paradigms, two-way transfer is not generally examined. Without a balanced and rigorous demonstration of bi-directional transfer, a skeptic can form a post-hoc argument that transfer effects are merely incidental side-effects of associative learning. Here, we tested for the transfer of serial knowledge in both directions.

Task performance was assessed in four different conditions. In two *novel* conditions, subjects learned lists in the SimChain or TI paradigms. In two *transfer* conditions, subjects first learned a list using one task, and later earned rewards performing the other task, using the ordering of stimuli they had initially learned. By comparing novel list learning to list transfer, we assessed their ability to transfer serial knowledge obtained from the first task to the second. Rather than rely on exhaustive training, subjects had no more than 160 trials in which to initially learn any list. Each condition required that subjects learn between 16 and 34 lists.

## Results

Subjects (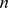 = 3) learned ordered lists composed of arbitrary photographic images in one of two paradigms: The SimChain paradigm [8], [18], [19], [23] and our counterbalanced TI paradigm. During the SimChain task, all of the list items were presented simultaneously on a touch-sensitive video monitor, with their configuration randomized on each trial. A food pellet was delivered after all of the items were selected in the correct sequence. The same lists were also trained using the TI task, in which only two items were presented on each trial. All possible pairs appeared in random order, counterbalanced such that each pair was presented *n* times before before any pair was presented *n*+1 times. A food pellet was delivered when the earlier of the two items was selected. See Materials & Methods for details about our procedure and our analytic methodology.

### Evidence of a Spatial Representation

**Figure 1:**
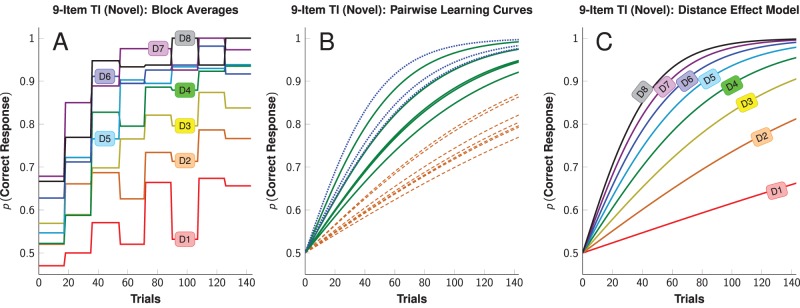
Learning function for the transitive inference (TI) task for one subject, Coltrane. “D1” corresponds to adjacent pairs, “D2” to pairs of items two positions apart, and so forth. *A*: Mean accuracy in 18-trial blocks, as a function of implicit distance between items. *B*: Logitistic regression model fit performed in isolation on each pair of distance 2 (orange dashed), distance 4 (green), and distance 6 (blue dotted). *C*: Logistic regression model fit for Equation 1 presented for each of the eight distances between items.

One advantage of our counterbalanced TI paradigm is that it enabled training of much longer lists than those used in most other studies of TI. Subjects learned 34 novel 9-item TI lists. Each list was trained in a single 144-trial session that consisted of four presentations of each of the 36 possible pairings. Subjects learned each list rapidly, despite only having a few opportunities to see each stimulus pairing. The rate of acquisition varied as a function of the distance between items. Figure 1A presents performance for Coltrane (a representative subject) in 18-response blocks, with pairs grouped by ordinal distance.

**Figure 2:**
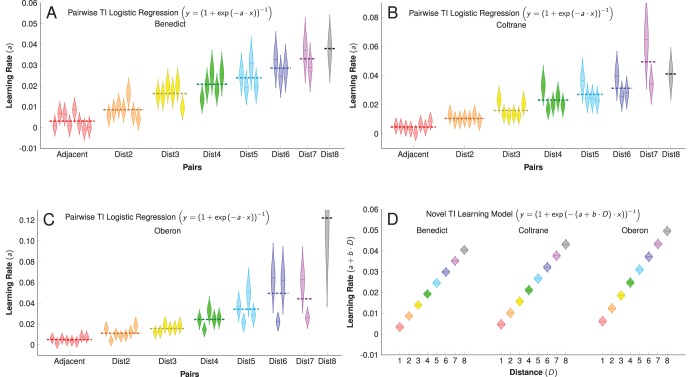
Slope parameters obtained from independent pairwise logistic regressions. Parameters are reported for Benedict (A), Coltrane, (B), and Oberon (C), as well as the compound slope from Equation 1 for all subjects (D). The ‘teardrop’ form of each point corresponds to the parameter’s probability density function over the 99% confidence interval. In general, larger parameters displayed correspondingly larger uncertainty.

We performed logistic regressions for the 36 stimulus pairings, each in isolation of the others, in order to predict accuracy as a function of trials. The slopes provide strong evidence for the SDE, despite being drawn from independent subsets of the data. Figure 1B shows Coltrane’s fitted functions for pairs spaced apart by two, four, and six ordinal ranks. Individual pairwise regression coefficients for each of our three subjects are shown in Figures 2A–C.

**Figure 3:**
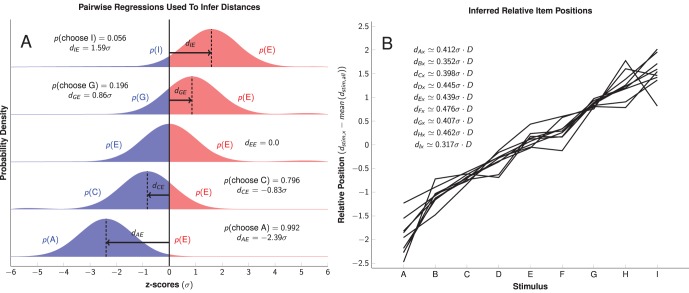
Method for inferring distance between items from pairwise logistic regressions, demonstrated using Coltrane’s parameters. *A*: Estimated probability of a correct response on the last trial of a session (based on the parameters from Figure 2B) is converted to a 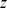-score using the normal inverse cumulative distribution. *B*: Comparison of relative item positions, based on inferred 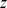-scores. Adjacent sitmuli were estimated to be separated by an average of 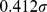 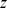-scores, arrayed along a linear continuum.

In order to make an inference about the distance between list items in a subject’s mental representation, we made the assumption that each item had a position on a linear continuum, with Gaussian error. If all error distributions had uniform variance, then a subject’s probability of making a correct response for any given pairing can be transformed into a *z*-score based on the cumulative normal density function, a process outlined in Figure 3A. This *z*-score provides a measure of the subjective distance between items. Using each item as a reference point, we obtained nine independent assessments of relative subjective distance. As shown in Figure 3B, the relative distances between items (as measured by error rates) were a linear function of their symbolic distance. Furthermore, those subjective distances were uniform regardless of which item was used as a reference point. Studies of numerical reasoning in monkeys have reported similar results [24].

Based on this linearity, we fit a logistic regression model in which pairs were pooled according to the ordinal distance between items (Equation 1, below). Figure 1C shows the fitted model of Coltrane’s performance as a function of trials and distance between items. Figure 2D presents the compound learning rate parameters for all subjects. Full regression statistics are provided in Information S1.

### Evidence of Transitive Inference during Initial Learning

To rule out the possibility that rote memorization was responsible for TI (particularly SDEs), we performed a logistic regression on only those trials falling in the first block of stimulus pairs for the novel 9-item lists (i.e. the first 36 responses). Thus, this subset of the data consisted of responses to pairs of stimuli never before seen by the subject. All subjects displayed a reliable SDE over this period (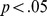 according to a Wald’s 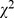 test for the coefficient 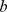). This result cannot be accounted for by memorization, and strongly implies that the transitive interrelationships between pairs were being integrated into their representations during this initial block of responses. The coefficients and other regression statistics are provided in Information S1.

### Transfer from SimChain to TI

To test for transfer from the SimChain task to the TI task, subjects were first trained for four consecutive days on a 5-item SimChain with elements A, C, E, G and I (4 sessions, 160 trials total). On the fifth day, subjects performed the TI task, using lists in which four unfamiliar items were interleaved among the five familiar ones, resulting in the 9-item list **A** *B* **C** *D* **E** *F* **G** *H* **I**. The odd-numbered list items (in bold) were previously learned in the SimChain task, while the even-numbered items (in italics) were unfamiliar. As a result, the stimulus pairs could fall into one of three categories: “familiar” pairs” (in which both were previously learned from the SimChain task), “unfamiliar pairs” (in which neither item had been seen previously), and “mixed” pairs (consisting of one familiar and one unfamiliar item).

**Figure 4:**
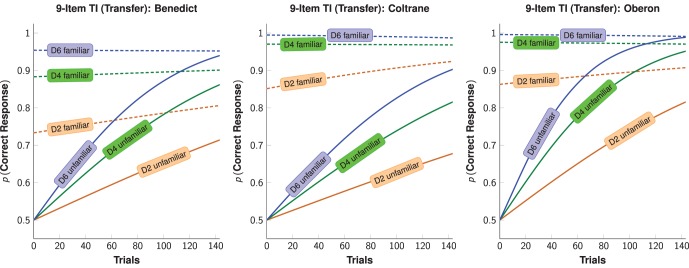
Learning functions for the TI task under the Transfer condition. These were based on the parameter fits for Equation 2, presented for familiar (dashed) and unfamiliar (solid) pairs.

We performed a logistic regression that included parameters for estimating accuracy on the very first trial (Equation 2, below). As can be seen in Figure 4, there was clear evidence of SDEs for both familiar and unfamiliar pairs (see Information S1). Accuracy to familiar pairs was nearly asymptotic on the first trial (albeit subject to the SDE), whereas accuracy to unfamiliar pairs increased gradually, as one would expect from trial and error learning.

### Transfer from TI to SimChain

**Figure 5:**
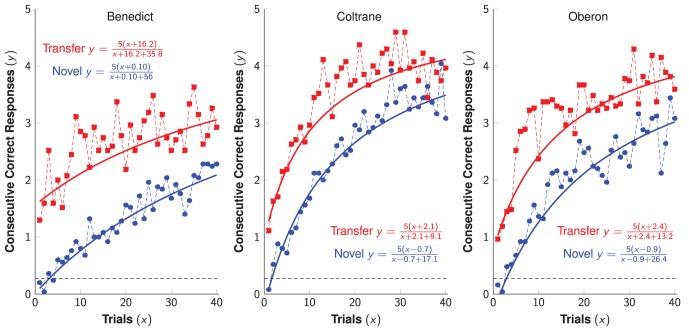
Number of correct responses before making an error in the SimChain task, as a function of trial and averaged across subjects. Chance responding is depicted as a dashed line.

To test for transfer from TI to SimChain, subjects first learned a 5-item list during a single session of TI training (120 trials) that consisted of twelve exposures to each of the ten possible pairings of items. Knowledge of that 5-item list was tested 24 hours later with a single session of SimChain (40 trials). Subjects repeated this process 25 times, each with a new list. Performance during the SimChain task was enhanced for transfer lists relative to novel lists. Figure 5 shows the mean number of consecutive response made without an error for novel lists (blue) and transfer lists (red). Also depicted is the level expected by chance, assuming no backwards errors (dashed black line). Backwards errors occurred on fewer than 1% of trials.

We fit a learning curve (Equation 3, below) to each subject’s data as a model of performance with respect to trial number and condition. The curve consisted of an effort parameter (corresponding to the speed of learning, with lower numbers being better) and a prior knowledge parameter (corresponding to the *y*-intercept). As compared to the novel condition, there was significant improvement for both parameters (Welch’s t-test, all 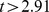, all 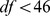, all 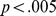). The most dramatic change was observed in the rate parameter. The learning rate was approximately twice as rapid in the transfer condition as in the novel condition. Further details are provided below, and in Information S1.

## Discussion

Subjects learned serial orderings using two paradigms. Although the demand characteristics of the SimChain and the TI tasks were qualitatively different, our subjects had no difficulty mastering them. This finding is consistent with previous results [23]. To demonstrate that both tasks made use of a common representation, we showed that prior experience with a list in one task enhanced performance in the other. In each instance, the benefits of transfer were visible from the first trial. We also observed symbolic distance effects (SDEs) in both novel and transfer conditions, supporting the claim that these distance effects arose not from the particular demands of each task, but rather from characteristics of their common representation. The learning curves we observed (Figure 2) support the hypothesis that list items were represented on a linear continuum (Figure 3).

Our data pose a difficulty for associative interpretations of the SDE because they assume that each item is associated with reinforcement and that distance effects were the result of contrasting associative strengths. Indeed, the “train adjacent first, test non-adjacent later” approach is intended to ensure that list items, other than the first and last, always have a 50% chance of being correct [13]. In this instance, there was no empirical support for the theoretical claim that differential reinforcement is a necessary and sufficient explanation of TI. Our methods (which ignored this concern) look identical to those obtained using traditional methods, suggesting that this concern is overblown.

Computational models that emulate properties of known neural networks have had considerable success in describing the specific patterns of learning in traditional TI tasks [14], [15], [17]. However, even though their designs are neurologically grounded, computational models have also been engineered to perform a narrow range of tasks. A neural network can be designed to approximate any behavioral output, but any given behavior could result from any one of a great many networks. Additionally, computational models often disregard the principle of parsimony [25]. Thus, while computational modeling qualifies as impressive engineering, the theoretical primacy of any one network has yet to be determined.

Differential reinforcement cannot easily account for performance in the SimChain task [26] because food pellets were only delivered after a subject responded to all items correctly. In designing a “perfect learning algorithm” for SimChain, a subject need only know the ordinal position of each item and the last item it touched. Nevertheless, when serial knowledge acquired from the SimChain task was used during the execution of the TI task, SDEs emerged in the differing asymptotic levels of performance that were observed in our present study (Figure 4), as well as in earlier experiments on one-way transfers from SimChain to TI [8].

It is not surprising that associationist models have struggled with (or ignored) the SimChain task. When all list items are visible throughout each trial, it is difficult to specify how credit for each reward should be assigned to the multiple stimuli that are simultaneously visible, unless one is willing to consider cognitive functions like memory or attention. This shortcoming renders the associative literature difficult to interpret outside of a handful of narrowly defined scenarios. Although associative models seem able to account for performance in the TI task, they are limited because they cannot adequately describe or predict SimChain performance [26]. This raises serious questions about claims regarding the parsimony of those models. A simpler model buys very little if it only explains a small range of scenarios.

Previous research has shown that SDEs can be obtained in one-way transfer experiments in which subjects were tested on two-item pairs that were selected from lists trained by the SimChain paradigm [8], [9], [27], as well as in traditional experiments that used the “train adjacent pairs, then test non-adjacent pairs” paradigm [13], [17], [18]. Our analysis showed that SDEs emerged during the first block of responding in the TI task, even though each of the possible pairs was presented only once in that portion of a session.

Our transfer experiments show that the experience of learning a serial task enhances performance from the first transfer trial on a qualitatively different task. Our results also support the hypothesis that the serial knowledge learned during the simultaneous chaining and the TI paradigms had the properties of a spatial representation reliant on relative item positions. Because SDEs need not arise from the task demands of the SimChain task, a common representation of list items provides a better explanation of error patterns than item associations. Figure 4 is especially informative in this regard because the SimChain paradigm does not require subjects to learn about non-adjacent pairs in order to execute the required sequence. Nevertheless, SDEs were obtained during the Transfer condition when subjects responded to familiar pairs. The consistency of SDEs and of transfer in both directions is all the more impressive when one considers that a 24-hour period separated the two tasks in all transfers we report.

The representation we propose is the most parsimonious mechanism for explaining serial learning and transfer of ordinal knowledge between distinct paradigms. The necessary parameters in this model are each item’s position on a linear continuum and the uncertainty associated with that position. These properties are can be captured by representing items as overlapping Gaussian distributions placed along that continuum (Figure 3).

In one sense, our proposal is conceptually compatible with an associative account. If we substitute “linear position” for “associative strength” and posit that associations display Gaussian error functions, then the difference between the models becomes semantic. In practice, it is associative *processes* that impose limits due to their strict reliance on rigid interpretations of “reinforcement.” Reinforcement learning models assert that associations only form when there is contiguity between behavior, the relevant stimuli, and the reinforcing outcome. Such models are constrained not by the possible forms that “associations” might have once learning has occurred, but rather by the narrow learning mechanisms that they are willing to consider. Although some argue that associative strength is sufficient to account for most results in the TI literature [13], most demonstrations of TI in animals use methodologies that are limited by associationism’s conceptual constraints. Since our subjects had no such difficulty learning lists using our two distinctive tasks, our results favor the cognitive account as providing a more general account of the observed phenomena.

### Serial Learning and Comparative Cognition

Our results are are best explained by a cognitive account, not only in terms of overall performance, but also with respect to observed patterns of error. Our spatial model permits better generalization across tasks than tailoring a custom equation for each task in isolation. It seems reasonable to conclude that our subjects “learned each list” in a general sense, rather than merely “learning each task” in the narrow sense of a circus trick.

The benefits of general representation are greatest when subjects must apply their knowledge to different tasks whose structural similarity is not immediately obvious from the surface features of each task. Traditionally, studies of human cognition have focused on analogical mechanisms to investigate how knowledge is applied across tasks [28]. Although humans can analogize in a conscious, deliberative fashion, there is no reason to suppose that this style of reasoning is available to non-human primates [2]. However, recent studies have demonstrated that humans also engage in entirely implicit analogical inferences in tasks requiring abstract serial cognition [29] and spatial processing [30]. These results closely resemble the generalization of serial knowledge we observed in rhesus macaques [27].

The systematic similarity of serial learning in human and non-human primates [3], [4], [31]–[33], and their mutual dissimilarity with more distantly related species, e.g., pigeons [32], suggests that the serial learning system underlying performance in SimChain and TI tasks is based on a cognitive mechanism that is common to primates. One possibility is that primates developed sophisticated serial representations to accommodate the increased complexity of their social structures. This hypothesis is consistent with evolutionary comparisons of multiple primate species in which transitive reasoning ability correlates with the social complexity typical of each species [34]. A convergent case has also been reported when comparing the transitive reasoning and social complexity of several species of corvids [35].

Recent advances in primate neurophysiology also shed considerable light on broad mechanisms underlying animal cognition. For example, parietal cortex, and particularly the lateral intraparietal area (LIP), is unambiguously implicated in spatial cognition [36]. Rather than merely encoding proximal stimulus information, these spatial representations are flexible, relying on reference to relative landmarks rather than mapping absolute position [37]. A growing body of electrophysiological work suggests that LIP is not merely spatial, but is instead involved in very general comparison-based reasoning, such as numerical reasoning [38] and comparisons of relative probability [39]. When analogous regions of the posterior parietal sulcus are examined in human subjects, selective activity is observed in TI tasks that can be dissociated from activity correlated with verbal processing [40].

Until recently, evidence of comparative cognition faced the criticism that “animal cognition” was an oxymoron. Such categorical rejections have become increasingly rare. Given unambiguous evidence of non-human cognitive processes, the next step is to assess how these processes apply to a variety of tasks, and to investigate their underlying mechanisms. In this paper, we have shown that monkeys can use a general cognitive mechanism to form serial representations applicable to two qualitatively different serial tasks. We hope that this demonstration will encourage further application of tools from human cognitive psychology to the study of similarities and differences in the cognitive mechanisms of humans and other animals.

## Materials and Methods

### Subjects

The subjects in our study were three rhesus macaques (*Macaca mulatta*), Benedict, Coltrane, and Oberon. All had prior experience using a touchscreen to earn food rewards. The daily food ration for these subjects was made available after they participated in our experiments.

This study was carried out in strict accordance with the recommendations in the Guide for the Care and Use of Laboratory Animals of the National Institutes of Health (NIH). This work was conducted at the Nonhuman Primate Facility of the New York State Psychiatric Institute with permission from its Department of Comparative Medicine’s (DCM) Institutional Animal Care and Use Committee (IACUC), protocol number 200, approved on 09/08/11, and with permission from the Columbia University IACUC, protocol number AC-AAAB1238, approved on 08/10/11.

Subjects were individually housed in rectangular Primate Products Enhanced Environment Housing, each with a nine-square-foot floorspace. Cages were maintained in colony rooms under 12-hour dark and light cycles, and the animals were given access to water ad libitum. Set amounts of Purina Monkey Chow (between 6 and 12 biscuits) and fruit were given after behavioral testing every day. The amounts of food dispensed were determined by the animals’ weight histories; weights were monitored on a weekly basis by research and veterinary staff to ensure subjects stayed at healthy weights. Subjects were given a variety of psychologically enriching tasks to complete at their discretion, beyond those required by behavioral testing. Primate Products enrichment mirrors, puzzle feeders, puzzles tosses, and kong toys were all provided to each individual in their cage; at least once a week, every subject was given sole access to an activity module containing additional kong toys and a prima-swing. No subject was physically harmed or knowingly exposed to potential infection.

### Apparatus

The apparatus consisted of a custom-built chamber with a touch-sensitive computer monitor mounted on one wall. This touch-screen both presented experimental stimuli and provided subjects with a user interface. Food rewards (Bioserve-brand pellets) were delivered to subjects in a receptacle that was located to the lower-left of the touchscreen. The apparatus was identical to that used in earlier experiments on monkey cognition [41].

### Procedure

Subjects completed sessions of TI and the SimChain as part of a daily battery of cognitive tasks. An ordered list of photographic stimuli provided the basis of the schedule for each session. Hereafter, we distinguish between item positions using letters, such as A for the first image of a list, B for the second, etc. The ordinal distance between any two items is denoted using the variable 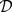 (not to be confused with the item D). For example, because the items in the adjacent pair BC were one rank apart, it follows that 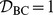. We also distinguished between *novel* lists, in which all stimuli were unfamiliar, and *transfer* lists, in which some or all of the stimuli had been presented in a previous session, preserving their original ordering.

Our SimChain task made use of 5-item lists. This task was identical to procedures described in previous studies [27], [41]. See [8] for review. Each session consisted of 40 trials.

The TI task used 5- or 9-item lists. During each trial, two images from the list were displayed on screen at random locations within a 4 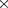 4 grid. The subject received a reward if it touched the image that came earlier in the list. Thus, a response to A was always rewarded, while responses to the last list item were never rewarded. Whether intermediate items were rewarded depended on the stimuli with which they were paired (touching C generated a reward when the pair CD was presented, but not in the case of the pair BC). A response to the image with a higher ordinal rank in the list was counted as an error, and resulted in a 6 second timeout.

There were ten possible response pairs in a 5-item list (AB, AC, AD, AE, BC, BD, BE, CE, CD, and DE), while a 9-item list entailed 36 possible pairs. TI sessions were divided into blocks, within which each pair of images was presented in a random order. Thus, each pair was presented *n* times before any pair was presented *n*+1 times. This counterbalanced the exposure that subjects received to each of the pairs with respect to overall session time. Sessions using 5-item lists had 12 blocks (in which each pair appeared 12 times, totaling 120 trials), while sessions using 9-item lists had 4 blocks (totaling 144 trials).

To test for transfer from TI to SimChain, subjects first completed a single session of TI, using a novel 5-item list. Twenty-four hours later, the same list was presented during a single session of SimChain. A total of 27 such transfers were executed. Performance on these transfer lists was compared to performance on 25 novel 5-item SimChain lists, also presented for only one session each.

To test for transfer from SimChain to TI, subjects completed four sessions of SimChain on consecutive days using a 5-item list consisting of the items ACEGI. On the fifth day, subjects completed a session of TI using the 9-item list, ABCDEFGHI. This 9-item transfer list consisted of both familiar items (A, C, E, G, and I, previously seen during the SimChain task) and unfamiliar items (B, D, F, and H, never used previously). Accuracy on 16 transfer lists was then compared with accuracy on 34 novel lists consisting of nine entirely unfamiliar items.

### Transitive Inference Analysis: Novel Lists

Accuracy in our transitive inference (TI) task was modeled using logistic regression as a way to interpolate across the different randomized orderings of pairs in each block of trials. Although this study alone does not provide sufficient data to build a comprehensive model of how information about item pairs is translated into a coherent representation, certain features of the representation can be inferred from patterns of acquisition.

Figure 1A shows the mean percentage of correct responses for the subject Coltrane in consecutive, non-overlapping 18-trial blocks, as a function of the distance 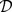 between list items (for example, since the pairs AC and BD are two ranks apart, 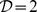 in both cases). Coltrane’s data were representative of the other subjects. Although these block averages intermix different pairs of stimuli, they nevertheless resemble the diminishing returns shape characteristic of a logistic function.

Figure 1A also shows a symbolic distance effect. Asymptotic accuracy was higher for more distant pairs, and distant pairs were also discriminated more rapidly. Despite nearly perfect accuracy to pairs like AG and BH after one session, subjects still made many errors when presented with adjacent pairs. Accuracy for adjacent pairs was well above chance, but not at a ceiling level.

By pooling data from the 34 novel 9-item lists learned by each subject, acquisition functions for each of the 36 distinct items pairs could be obtained using the logistic regression 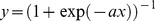. No constant was included because subjects were presumed to initially respond at chance levels. Figure 1B shows fitted curves of Coltrane’s performance for each of the pairs of distance 2 (dashed orange), 4 (green), and 6 (dotted blue). Despite being drawn from independent subsets of the data, the learning rate for each pair appears to be consistently related to the distance between items. This is confirmed by an examination of the regression coefficients obtained for all 36 pairs, presented in Figure 2A (Benedict), Figure 2B (Coltrane), and Figure 2C (Oberon).

Given the consistency with which the learning curve was predicted by distance 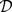, we fit the following logistic regression as a model of novel TI responding:
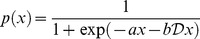
(1)


Here, the probability of success 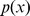 on trial 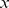 is determined both an overall learning rate parameter 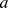 (the ‘general’ learning rate) and a distance-related learning rate parameter 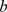 that is multiplied by that pair's ordinal distance 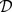 (the ‘distance-specific’ learning rate). Figure 1C shows the model fit for Equation 1 given Coltrane’s data, while Figure 2D presents each subject’s compound learning rate as a function of 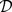.

### Evidence For A Spatial Representation

Given the apparent consistency with which the ordinal distance between items predicted the learning rate, we sought to determine whether the pairwise regression parameters presented in Figure 2 could be used to infer the confusability of stimulus pairs and, following the logic of basic signal detection, the distance between items.

Each of the pairwise logistic regression models was used to estimate the probability of choosing an item at the end of the first session of learning (that is, on trial 144). For example, in the case of the pair AE, the pairwise logistic regression of Coltrane’s data suggests a probability of 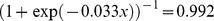. This probability was converted to a 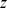-score based on the normal inverse cumulative distribution. Thus, the subjective distance from item E to item A, denoted by 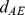, was estimated to be 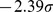. Figure 3A shows how this was applied to four of Coltrane’s 36 pairs. The hypothetical pair EE is also depicted to show how chance performance would look in the case where subjects would be presented with two identical stimuli.

Nine subjective distances 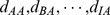 were estimated using independently obtained regression coefficients of each pair including the item A, and these were compared as a factor of the ordinal distance between items 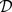. A similar comparison was made using each of the eight other stimuli as a reference point. The relative distance between these items was highly consistent, as displayed in Figure 3B for Coltrane’s data. The subjective distances inferred from the observed symbolic distance effects resulted in linear relationships for all nine items, each displaying a similar slope. This means that subjects consistently responded as though items were arrayed at consistently-spaced intervals along a linear continuum, with overlapping Gaussian uncertainty about their position.

### Transitive Inference Analysis: Transfer Lists

In order to examine the effects of transferring list knowledge from the SimChain task, a more complex logistic regression was required:
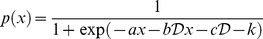
(2)


This model was identical to Equation 1, except for the addition of two additional parameters that allowed the model intercept to begin at a value other than 0.5. These included a general constant 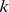 that allowed responding to begin above chance generally, and a distance-influenced constant 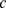 that changed the intercept as a function of the ordinal distance between list items 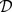.

Figure 4 shows the model fit parameters for familiar pairs (dashed lines) and unfamiliar pairs (solid lines) at three different pair distances for each of the subjects. Two key conclusions can be drawn from these model fits. The first is that transfer unambiguously occurred, with subjects responding at asymptotic levels to the familiar pairs despite being naïve about unfamiliar pairs. The other is that the familiar pairs displayed a symbolic distance effect, despite the fact that those items were learned by performing the SimChain task (in which learning about non-adjacent relationships is not required).

### Simultaneous Chain Analysis

In order to obtain a descriptive model for SimChain learning, we used the following learning curve equation [42]:
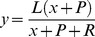
(3)


Here, the performance 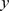 (whose minimum is 0.0 and whose maximum is 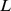) is described as a function of 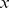 trials, prior knowledge 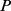, and learning cost 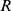, where lower values of 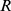 correspond to faster learning. If 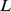 is unknown, the best-fitting parameters must be determined numerically. However, because our SimChain task always made use of 5-item lists, 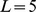 in all subsequent computations. This permits straightforward parameter estimation. The resulting model fits are depicted in Figure 5.

The learning cost 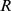 is best understood in terms of the cost in time of making additional progress. The first 50% of performance (over the range 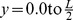) is expected to take 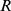 trials, and each additional 50% improvement should take twice again as long. Thus, if 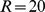, it would take 20 trials to go from 0% to 50% accuracy, an additional 40 trials to go from 50% to 75% accuracy, an additional 80 trials to go from 75% to 87.5% accuracy, etc., with performance reaching 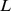 at asymptote.

Prior knowledge 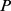 indicates the benefit of knowledge that was obtained before to the first trial. Thus, if 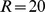 and 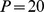, performance will begin at 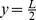. The value of 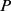 is relative to the value of 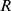.

When 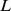 is known, estimating the parameters 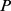 and 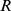 can be converted to a linear form:
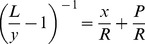
(4)


Since it is known that 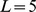, we can compute the slope 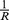 and the intercept 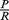 using a regression model. Because of the transformation of the dependent measure, ordinary least squares regression is inappropriate. The variance of 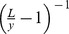 grows in an approximately exponential fashion as 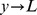. We can then fit Equation 4 using weighted least squares, with the inverse of the sample variance as weights [43].

## Supporting Information

Information S1 **Regression tables.** Supplemental tables providing full regression statistics for all models in the study.
(PDF)
