# Supplementary material for: Transfer of a Serial Representation between Two Distinct Tasks by Rhesus Macaques
Source: PLoS One. 2013 Jul 31;8(7):e70285. doi: 10.1371/journal.pone.0070285 (PMC3729468; doi:10.1371/journal.pone.0070285)
Supplement: Information S1 — Regression tables. Supplemental tables providing full regression statistics for all models in the study. (PDF) [file pone.0070285.s001.pdf]

This supplement provides the regression statistic for the analyses reported in the main text. Note that all Wald's χ^2 ($df = 1$), All Model χ^2 ($df = 2$)

Table S1: **Novel 9-Item TI Lists (Logistic Regression)**

Benedict						
Coeff	β	SE β	Wald's χ^2	p	Model χ^2	p
a	-0.0019	0.0008	4.89	$< .03$	502.76	$< .0001$
b	0.0053	0.0003	298.70	$< .0001$		
Coltrane						
Coeff	β	SE β	Wald's χ^2	p	Model χ^2	p
a	-0.0008	0.0009	0.86	.35	569.66	$< .0001$
b	0.0055	0.0003	290.06	$< .0001$		
Oberon						
Coeff	β	SE β	Wald's χ^2	p	Model χ^2	p
a	-0.0015	0.0009	3.00	.08	590.07	$< .0001$
b	0.0062	0.0003	324.02	$< .0001$		

Logistic regressions on novel 9-item TI lists, corresponding to Equation 1 and to the model fits plotted in Figures 1C and 2D.

Table S2: **Transfer 9-Item TI Lists, Familiar Pairs (Logistic Regression)**

Benedict

	Full Model				Reduced Model			
Coeff	β	SE β	Wald's χ^2	p	β	SE β	Wald's χ^2	p
k	-0.3148	0.5492	0.33	.56	0	–	–	–
c	0.5935	0.1725	11.83	$< .0006$	0.5054	0.0715	49.99	$< .0001$
a	0.0078	0.0067	1.37	.25	0.0045	0.0033	1.80	.18
b	-0.0017	0.0020	0.68	.41	-0.0008	0.0012	0.37	.54
	Model $\chi^2 = 44.60$			$< .0001$	Model $\chi^2 = 44.27$			$< .0001$

Coltrane

	Full Model				Reduced Model			
Coeff	β	SE β	Wald's χ^2	p	β	SE β	Wald's χ^2	p
k	0.9498	0.8477	1.26	.26	0	–	–	–
c	0.5568	0.2907	3.37	.06	0.8721	0.1278	46.53	$< .0001$
a	0.0011	0.0106	0.01	.93	0.0111	0.0054	4.17	$< .05$
b	0.0003	0.0037	0.01	.92	-0.0029	0.0022	1.81	.17
	Model $\chi^2 = 23.05$			$< .0002$	Model $\chi^2 = 21.84$			$< .0001$

Oberon

	Full Model				Reduced Model			
Coeff	β	SE β	Wald's χ^2	p	β	SE β	Wald's χ^2	p
k	0.2568	0.9799	0.07	.79	0	–	–	–
c	0.8272	0.3738	4.90	$< .03$	0.9195	0.1415	42.83	$< .0001$
a	0.0048	0.0118	0.17	.68	0.0075	0.0058	1.64	.20
b	-0.0017	0.0044	0.09	.77	-0.0022	0.0025	0.82	.37
	Model $\chi^2 = 29.47$			$< .0001$	Model $\chi^2 = 29.41$			$< .0001$

Logistic regressions on the unfamiliar items from transfer 9-item TI lists, corresponding to Equation 2 and to the model fits plotted in Figures 4.

Table S3: **Transfer 9-Item TI Lists, Mixed Pairs (Logistic Regression)**

			Benedict					
	Full Model				Reduced Model			
Coeff	β	SE β	Wald's χ^2	p	β	SE β	Wald's χ^2	p
k	0.0645	0.2161	0.09	.56	0	–	–	–
c	0.2372	0.0693	11.71	$< .0006$	0.2541	0.0401	40.06	$< .0001$
a	-0.0013	0.0027	0.23	.25	-0.0006	0.0014	0.19	.66
b	0.0016	0.0009	3.03	.41	0.0014	0.0007	4.35	$< .04$
	Model $\chi^2 = 103.39$			$< .0001$	Model $\chi^2 = 103.30$			$< .0001$
			Coltrane					
	Full Model				Reduced Model			
Coeff	β	SE β	Wald's χ^2	p	β	SE β	Wald's χ^2	p
k	0.1052	0.2284	0.21	.26	0	–	–	–
c	0.3485	0.0787	19.58	.06	0.3779	0.0460	67.52	$< .0001$
a	0.0025	0.0028	0.79	.93	0.0036	0.0014	6.50	$< .02$
b	-0.0004	0.0010	0.20	.92	0.0007	0.0007	1.20	.27
	Model $\chi^2 = 71.13$			$< .0002$	Model $\chi^2 = 76.92$			$< .0001$
			Oberon					
	Full Model				Reduced Model			
Coeff	β	SE β	Wald's χ^2	p	β	SE β	Wald's χ^2	p
k	0.1941	0.2205	0.77	.79	0	–	–	–
c	0.0056	0.0688	0.01	$< .03$	0	–	–	–
a	-0.0022	0.0030	0.54	.68	0	–	–	–
b	0.0067	0.0012	31.04	.77	0.0069	0.0005	227.03	$< .0001$
	Model $\chi^2 = 164.88$			$< .0001$	Model $\chi^2 = 162.18$			$< .0001$

Logistic regressions on the 'mixed' items from transfer 9-item TI lists, corresponding to Equation 2.

Table S4: **Transfer 9-Item TI Lists, Unfamiliar Pairs (Logistic Regression)**

Benedict								
	Full Model				Reduced Model			
Coeff	β	SE β	Wald's χ^2	p	β	SE β	Wald's χ^2	p
k	-0.0826	0.5222	0.03	.87	0	–	–	–
c	0.1430	0.1487	0.93	.33	0	–	–	–
a	-0.0010	0.0020	0.02	.88	0	–	–	–
b	0.0021	0.0066	1.19	$< .03$	0.0032	0.0004	51.84	$< .0001$
	Model $\chi^2 = 18.17$			$< .002$	Model $\chi^2 = 13.62$			$< .0003$

Coltrane								
	Full Model				Reduced Model			
Coeff	β	SE β	Wald's χ^2	p	β	SE β	Wald's χ^2	p
k	-0.2649	0.5213	0.26	.61	0	–	–	–
c	0.1640	0.1465	1.25	.26	0	–	–	–
a	0.0017	0.0066	0.07	.79	0	–	–	–
b	0.0011	0.0019	0.35	.55	0.0026	0.0004	39.56	$< .0001$
	Model $\chi^2 = 14.41$			$< .007$	Model $\chi^2 = 11.28$			$< .0008$

Oberon								
	Full Model				Reduced Model			
Coeff	β	SE β	Wald's χ^2	p	β	SE β	Wald's χ^2	p
k	-0.6519	0.5598	1.36	.24	0	–	–	–
c	0.2333	0.1643	2.02	.16	0	–	–	–
a	0.0046	0.0079	0.35	.56	0	–	–	–
b	0.0033	0.0026	1.63	.20	0.0052	0.0006	75.06	$< .0001$
	Model $\chi^2 = 40.33$			$< .0001$	Model $\chi^2 = 37.47$			$< .0001$

Logistic regressions on the unfamiliar items from transfer 9-item TI lists, corresponding to Equation 2 and to the model fits plotted in Figures 4.

Table S5: **Novel 9-Item TI Lists, First Block Only (Logistic Regression)**

Benedict						
Coeff	β	SE β	Wald's χ^2	p	Model χ^2	p
a	-0.0138	0.0059	2.32	.13	67.10	$< .0001$
b	0.0120	0.0018	6.86	$< .01$		
**Coltrane**						
Coeff	β	SE β	Wald's χ^2	p	Model χ^2	p
a	-0.0054	0.0057	0.95	.33	36.73	$< .0001$
b	0.0077	0.0016	4.94	$< .03$		
**Oberon**						
Coeff	β	SE β	Wald's χ^2	p	Model χ^2	p
a	-0.0170	0.0058	2.94	.08	74.55	$< .0001$
b	0.0129	0.0017	7.39	$< .007$		

Logistic regressions on only the first block of items novel 9-item TI lists, using Equation 1, as reported in the results section entitled 'Evidence of Transitive Inference During Initial Learning.'

Table S6: **SimChain Performance, Weighted Linear Regression**

Benedict								
	Novel				Transfer			
Coeff	β	SE β	$t(38)$	p	β	SE β	$t(38)$	p
$\frac{P}{R}$	0.0019	0.0080	0.241	.81	0.4523	0.0584	7.741	$< .0001$
$\frac{1}{R}$	0.0179	0.0001	20.103	$< .0001$	0.0279	0.0036	7.721	$< .0001$
P	0.10				16.19			
R	56.00				35.79			
**Coltrane**								
	Novel				Transfer			
Coeff	β	SE β	$t(38)$	p	β	SE β	$t(38)$	p
$\frac{P}{R}$	-0.0413	0.0060	-6.916	$< .0001$	0.2310	0.0465	4.962	$< .0001$
$\frac{1}{R}$	0.0587	0.0020	28.63	$< .0001$	0.1104	0.0080	13.787	$< .0001$
P	-0.70				2.10			
R	17.05				9.07			
**Oberon**								
	Novel				Transfer			
Coeff	β	SE β	$t(38)$	p	β	SE β	$t(38)$	p
$\frac{P}{R}$	-0.0337	0.0123	-2.7476	.009	0.1900	0.0445	4.266	$< .0001$
$\frac{1}{R}$	0.0378	0.0020	18.4867	$< .0001$	0.0796	0.0059	13.486	$< .0001$
P	-0.89				2.41			
R	26.43				13.18			

Weighted learning curve regressions for 5-item SimChain, corresponding to Equation 4 and to the model fits plotted in Figures 5.

Table S7: **Differences Between Novel and Transfer Parameters**

	Benedict			Coltrane			Oberon		
Coeff	t	df	p	t	df	p	t	df	p
$\frac{P}{R}$	7.43	39.18	$< .0001$	5.81	39.26	$< .0001$	4.41	43.85	$< .0001$
$\frac{1}{R}$	2.91	41.59	$< .006$	6.26	42.99	$< .0001$	6.95	45.66	$< .0001$

Weighted regression parameter comparisons for the learning curve regressions from Table S6.
